# Quantitative proteomics of formalin-fixed, paraffin-embedded cardiac specimens uncovers protein signatures of specialized regions and patient groups

**DOI:** 10.1038/s44161-025-00721-2

**Published:** 2025-09-26

**Authors:** Jonathan S. Achter, Thomas H. L. Jensen, Paola Pisano, Johan S. Bundgaard, Daniel Raaschou-Oddershede, Kasper Rossing, Michael Wierer, Alicia Lundby

**Affiliations:** 1 Department of Biomedical Sciences, Faculty of Health and Medical Sciences, University of Copenhagen, Copenhagen, Denmark; 2 Department of Pathology, Diagnostic Center, Copenhagen University Hospital, Copenhagen, Denmark; 3 Proteomics Research Infrastructure, Faculty of Health and Medical Sciences, University of Copenhagen, Copenhagen, Denmark; 4 Department of Cardiology, The Heart Center, Copenhagen University Hospital, Copenhagen, Denmark; 5 Department of Clinical Immunology, Copenhagen University Hospital, Copenhagen, Denmark; 6 Department of Clinical and Translational Research, Steno Diabetes Center Copenhagen, Herlev, Denmark

**Keywords:** Proteomic analysis, Cardiovascular biology, Cardiomyopathies, Systems analysis

## Abstract

Proteomic technologies have advanced our understanding of disease mechanisms, patient stratification and targeted therapies. However, applying cardiac proteomics in translational research requires overcoming the barrier of tissue accessibility. Formalin-fixed, paraffin-embedded (FFPE) heart tissue, widely preserved in pathology collections, remains a largely untapped resource. Here we demonstrate that proteomic profiles are well preserved in FFPE human heart specimens and compatible with high-resolution, quantitative analysis. Quantifying approximately 4,000 proteins per sample, we show this approach effectively distinguishes disease states and subanatomical regions, revealing distinct underlying protein signatures. Specifically, the human sinoatrial node exhibited enrichment of collagen VI and G protein-coupled receptor signaling. Myocardial biopsies from patients with arrhythmogenic cardiomyopathy were characterized by fibrosis and metabolic/cytoskeletal derangements, clearly separating them from donor heart biopsies. This study establishes FFPE heart tissue as a robust resource for cardiac proteomics, enabling retrospective molecular profiling at scale and unlocking archived specimens for disease discovery and precision cardiology.

## Abstract (web summary)

Achter et al. established a protocol for quantitative proteomic profiling of formalin-fixed, paraffin-embedded human cardiac tissues, benchmarked against fresh-frozen samples. They applied it to stratify patients with arrhythmogenic cardiomyopathy and performed deep proteomic analysis of the human sinoatrial node.

## Main

Over the past two decades, we have witnessed unprecedented progress in elucidating the etiologies of cardiovascular diseases, driven by the advent of omics technologies. The mapping of disease associations for numerous genetic variants has revealed the complex genetic architecture of these conditions^1–3^. While genomics has transformed clinical research and diagnostics, additional molecular methods are essential to elucidate the functional consequences and their relevance. Moreover, complementary methods are needed to investigate diseases attributed to the cumulative effects of life-long exposure to environmental and lifestyle risk factors. Proteomics studies represent a powerful strategy to evaluate the integrated effects of genetic variants as well as external risk factors, thereby making it an attractive strategy to address underpinnings of multifactorial diseases, such as those of the cardiovascular system^4^. Ultimately, precision medicine depends on the ability to distinguish patient subgroups based on molecular profiles to tailor therapies effectively. Advancements in omics, particularly driven by cancer research, have led to substantial progress in our understanding of molecular heterogeneity and enabled the stratification of patients. For example, molecular characterization has effectively grouped patients with breast cancer for tailored treatment^5–7^. However, translating such progress to cardiovascular disease remains challenging, partly due to the difficulty of accessing heart tissue for large-scale investigations of disease-specific molecular profiles.

In clinical settings, cardiac specimens collected for diagnostic purposes are commonly preserved as formalin-fixed, paraffin-embedded (FFPE) tissue samples. For decades, such cardiac FFPE samples have enabled detailed histopathological analyses, forming the backbone of diagnostic and prognostic assessments, for instance, in evaluations of suspected autoimmune disease in patients with unexplained heart failure, giant cell myocarditis, eosinophilic myocarditis, vasculitis or sarcoidosis^8^. Archives of FFPE preserved cardiac specimens in hospital pathology sections thus represent an invaluable resource, overcoming barriers to tissue acquisition for large cohort investigations and enabling molecular profiling across diverse (sub)groups of patients with cardiac disease.

Mass spectrometry-based proteomics has emerged as the method of choice for deep and unbiased quantitative investigations of the proteome. While proteomic studies of FFPE samples have gained momentum in fields like oncology, quantifying thousands of proteins, similar work in cardiology has lagged significantly behind. Previous studies on human heart proteomes were essentially limited to fresh-frozen (FF) tissue samples^9–13^, shallow protein coverage^14,15^ or cell-line-based studies^16^. Inspired by the recent proteomic advancements in other fields^17–19^, we set out to evaluate whether a similar strategy could characterize the proteomes of human heart FFPE samples.

**Fig. 1:**
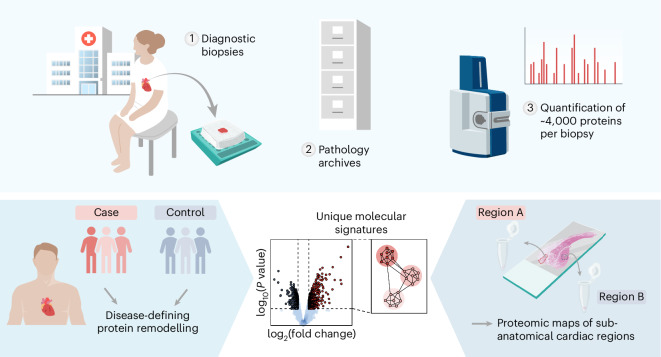
Comprehensive proteomic profiling of cardiac FFPE tissue specimens. Upper panel: Overview of the study approach, in which diagnostic cardiac biopsies (1) preserved as FFPE blocks in pathology archives (2) are analyzed by high-resolution mass spectrometry to quantify approximately 4,000 proteins per sample (3). Lower panel: Two example applications of this approach. Left: comparison of protein profiles between patient cases and controls to identify disease-defining patterns of protein remodeling. Right: generation of proteomic maps for distinct sub-anatomical cardiac regions to reveal spatially resolved molecular signatures.

Formalin fixation preserves tissue through extensive protein crosslinking, complicating subsequent proteomic analyses^20^. A key objective of the present study was to benchmark the comparability of proteomic data from FF versus FFPE cardiac tissue samples. By quantifying the variation introduced by formalin fixation relative to biological and technical sources, we establish the reliability of cardiac FFPE tissue for quantitative proteomics. We present two case studies to illustrate this approach’s potential for studying human heart samples collected in a diagnostic context and stored as FFPE preserved samples in a hospital archive (Fig. 1). First, we showcase spatially relevant proteomic mapping in cardiac tissue, addressing regional specializations. Second, we apply the approach to disease profiling in patients with arrhythmogenic cardiomyopathy (ACM), exemplifying proteomics-driven disease characterization. This study highlights the potential of deep proteomics investigations of FFPE heart tissue samples to advance both fundamental and applied cardiac research, establishing a robust framework for future proteomic studies and paving the way for retrospective proteomics in clinical and translational cardiac research.

## Results

### Preserved cardiac proteome profiles are retrieved from FFPE heart tissue samples

**Fig. 2:**
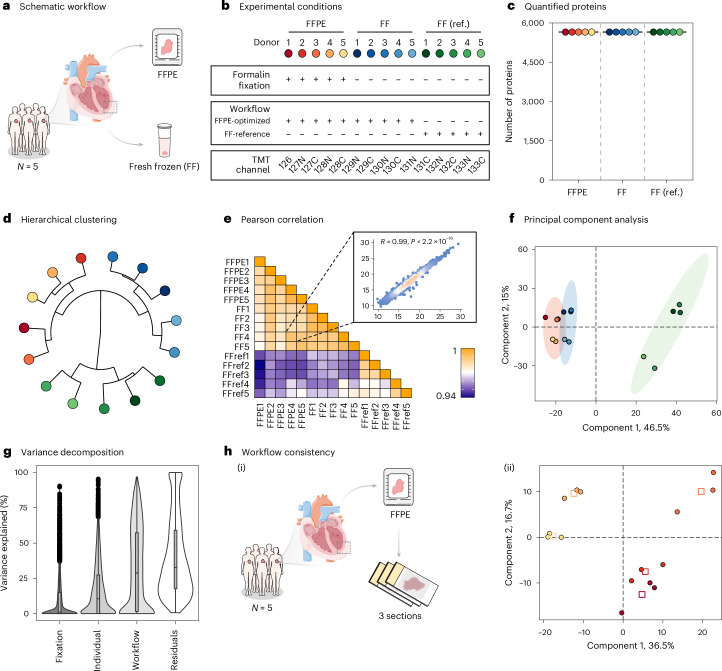
Comparative proteomic analysis of FFPE and FF cardiac tissue. **a**, Schematic representation of the study design. Paired FFPE and FF tissue samples were collected from the myocardium of five human hearts for proteomic evaluation (*N* = 5). **b**, Overview of experimental conditions evaluated using a TMT-multiplexed strategy, resulting in 15 samples. **c**, Number of proteins quantified across FFPE, FF and reference (FF ref.) workflows. **d**, Hierarchical clustering dendrogram of protein expression profiles. **e**, Pearson correlation heat map depicting pairwise correlation coefficients. The inset scatter plot illustrates representative sample correlation. **f**, PCA of the 15 samples based on their protein expression profiles. The PC1 and PC2 account for 46.5% and 15% of the variance, respectively. Ellipses indicate 90% confidence intervals for each group. **g**, Variance decomposition of all quantified proteins, showing contributions of fixation, individual differences, workflow and residual variability. Box plots depict medians (center lines), interquartile ranges (IQR; boxes) and values within 1.5× the IQR (whiskers); points outside this range are plotted. **h**, Workflow consistency analysis of FFPE samples (*N* = 15; three sections per five FFPE blocks): (i) schematic of sectioning and (ii) discriminant analysis of resulting proteome profiles, color coded per individual. Squares mark the centroids of replicate sections. Parts of panel **a** adapted from Servier Medical Art (https://smart.servier.com/) under a Creative Commons license CC BY 4.0. Source data

**Extended Data Fig. 1:**
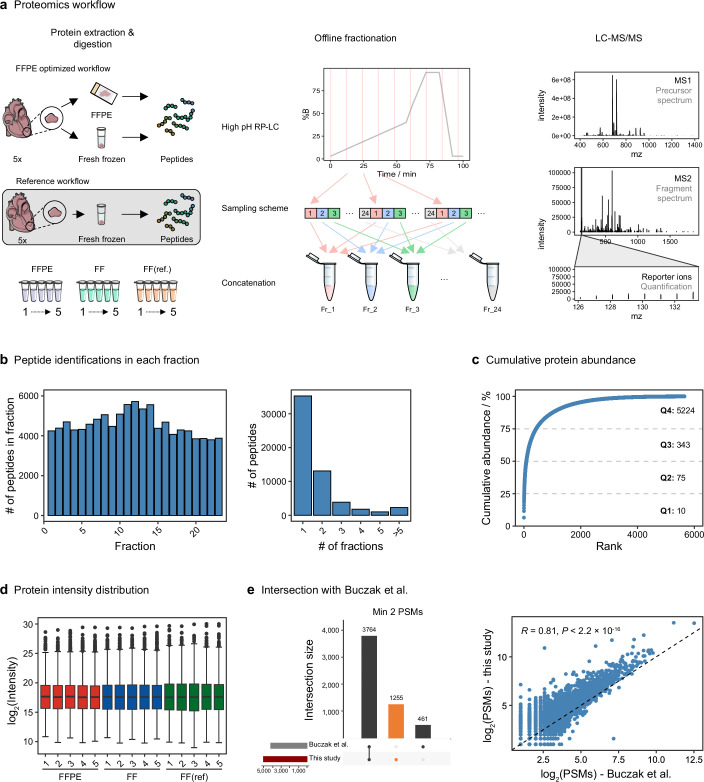
TMT-based proteome profiling of FFPE and FF cardiac tissue. (**a**) Schematic of the workflow used for proteome analysis of FFPE and FF cardiac tissues. Proteins were extracted and digested using the FFPE-optimized and reference workflows, followed by TMT labeling and peptide separation by high-pH reversed-phase liquid chromatography (RP-LC) off-line fractionation. Examples of MS1 precursor and MS2 fragment spectra illustrate the detection and quantification of reporter ions. (**b**) Peptide identifications in fractions. Left: Distribution of identified peptides across all fractions. Right: Histogram showing the number of fractions in which peptides were detected. (**c**) Cumulative protein abundance. Proteins are ranked by summed TMT reporter ion intensities, with protein counts indicated for each quartile (Q1–Q4). (**d**) Boxplot representation of protein intensities across all multiplexed samples, showing the median (center line), interquartile range (box), and whiskers extending to 1.5× the IQR. Outliers are shown as individual points. (**e**) Intersection with a reference dataset. Left: UpSet plot depicting the overlap of proteins quantified by at least two peptide spectrum matches (PSMs) in this study with those reported by Buczak et al.^22^ Right: Scatter plot of PSM counts per protein, comparing results from this study with those from Buczak et al. Spearman correlation coefficient (R; two-sided p-value). Source data

To assess the feasibility of deep quantitative proteomic profiling from human heart FFPE biospecimens, we designed a controlled study to determine whether the cardiac proteome is preserved and can be retrieved from these samples. We pursued a direct comparison of proteomic profiles from human heart tissue that was either FF or FFPE preserved. Left ventricular tissue was obtained from five human hearts, with each sample split into two: one part was flash-frozen, and the other part formalin-fixed and paraffin-embedded (Fig. 2a). To evaluate the impact of processing steps required to reverse formalin-induced protein crosslinking and enable effective removal of paraffin, protein extraction was pursued by three distinct approaches (Extended Data Fig. 1a): (1) an optimized workflow for FFPE tissue, designed to address crosslinking and efficiently process paraffin-containing scrolls without the use of highly toxic xylene^21^, (2) the same optimized workflow applied to FF samples to isolate the effect of tissue preservation and (3) an established protocol for FF samples used as a benchmark for comparison^11^.

The extracted proteins were digested, and the resulting peptides were multiplexed using tandem mass tags (TMT) for relative quantification across all samples (Fig. 2b). To maximize proteome coverage, we performed fractionation at high pH to reduce sample complexity before high-resolution liquid chromatography-tandem mass spectrometry (LC–MS/MS) measurements. We measured 24 individual fractions, identifying a total of 5,915 proteins, 57,330 peptides and 218,400 peptide spectrum matches (PSMs) (Extended Data Fig. 1a,b). Of these, 5,697 unique proteins were quantified based on the sample-specific reporter ions in the MS/MS spectra (Fig. 2c and Extended Data Fig. 1c,d). It is worth noting that 5,652 proteins were quantified with no missing values, achieving over 99% data completeness. Compared to previous efforts using a similar approach^22^, we identified 2.3 times more PSMs, 1.5 times more peptides and 1.2 times more proteins (Extended Data Fig. 1e). Despite the inherent dynamic range challenge of heart proteomes, where a small subset of proteins disproportionately contributes to the overall signal (Extended Data Fig. 1c), each protein was quantified on average by 18 PSMs. This provides confidence in the relative protein quantifications obtained.

**Extended Data Fig. 2:**
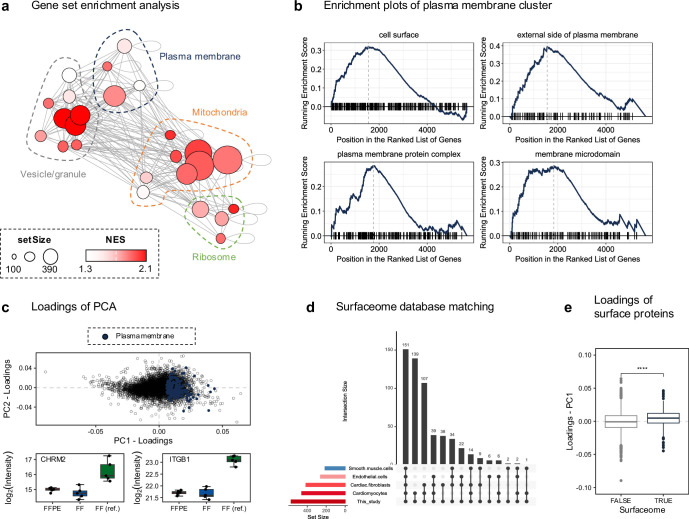
Workflow-dependent proteomic variation in FFPE and reference cardiac samples. (**a**) Clustering of positively enriched gene sets along PC1, based on term similarity, reveals distinct functional themes. (**b**) Enrichment plots for gene sets within the plasma membrane cluster. (**c**) PCA loadings. Top: Proteins core-enriched in the plasma membrane cluster are highlighted in the PC1-PC2 plot. Bottom: Representative boxplots showing abundance of CHRM2 (left) and ITGB1 (right). Boxplots are based on N=5 biological replicates per group and display the median (center line), interquartile range (box), and whiskers extending to 1.5× the IQR; measurements are shown as individual points. (**d**) UpSet plot showing the intersection between proteins identified in this study and those in the cardiac surfaceome database^23^. (**e**) PC1 loadings of proteins present in the surfaceome database versus all other proteins. Each boxplot displays the median (center line), interquartile range (box), and whiskers extending to 1.5× the interquartile range (IQR). Outliers beyond the whiskers are shown as individual points. Statistical significance was assessed using a two-sided Wilcoxon rank-sum test (P = 1.39 × 10^−17^). Source data

Hierarchical clustering of measured protein intensities grouped samples according to the three experimental conditions evaluated, showing that FFPE and FF samples processed with the same workflow were more similar to each other than to FF samples processed with the reference workflow (Fig. 2d). Pearson correlation analysis highlighted the excellent reproducibility in protein intensity measurements across all samples (*R* > 0.94), with FFPE and FF samples processed with the same workflow showing particularly high correlation (*R* = 0.99) (Fig. 2e). Principal component analysis (PCA) indicated that the primary source of variation was the tissue processing workflow rather than preservation strategy (Fig. 2f). Inspired by the noted differences in protein extraction, we evaluated biases among proteins contributing to this difference. Gene set enrichment analysis (GSEA) of first principal component (PC1) loadings showed enrichment in membrane-enclosed organelle proteins, among other plasma membrane proteins, in reference FF samples (Extended Data Fig. 2a–c). Intersection with a cardiac cell surface protein atlas^23^ supported that membrane proteins significantly contribute to this separation (Extended Data Fig. 2d,e). This suggests that, while relative protein quantification is consistent across all replicates within a given condition, the retrieval of proteins from specialized compartments, such as the plasma membrane, is more challenging from FFPE samples. The PCA also highlighted that the second principal component (PC2) captured biological differences between individuals (1 + 2 + 3 versus 4 + 5) (Fig. 2f), which encouraged us to explore sources of variation in the dataset further. Variance decomposition analysis showed that the sample processing workflow accounted for a median of 28.9% of proteome-wide variance, followed by subject differences at 10.6%, while formalin fixation of the samples contributed minimally at just 1.1% (Fig. 2g). These results support the ability of the approach to capture inter-individual differences with minimal influence from fixation on quantitative profiles.

Finally, to assess our ability to capture inter-individual differences from the cardiac proteomes and workflow reproducibility, we collected three sections from each of the five paraffin-embedded hearts (Fig. 2h). A subsequent TMT-multiplexed proteomic profiling experiment quantified 4,998 unique proteins without any missing values (91% overlap with previous measurements) based on 80,523 PSMs. Partial least-squares discriminant analysis of the obtained dataset efficiently distinguished the biological measurements of individual samples, with tight clustering of the three replicates per individual (Fig. 2h). These results confirm that consistent, sample-specific cardiac proteome profiles can be obtained from FFPE specimens.

Collectively, the above results show that the choice of preservation has minimal impact on relative protein abundances. FFPE cardiac proteome profiling is well suited for relative quantitative investigations and is on par with current techniques used for FF heart tissue.

### Scaling throughput of cardiac proteome profiling with label-free data-independent acquisition

**Fig. 3:**
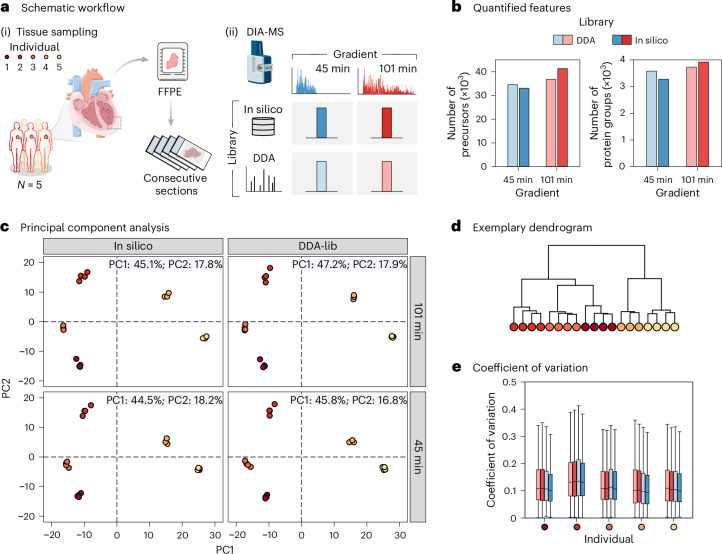
Evaluation of DIA-MS acquisition strategies using in silico and DDA-based libraries for proteomic profiling of FFPE heart tissue. **a**, Schematic workflow: (i) Tissue sampling and preparation from FFPE cardiac specimens of five individuals (*N* = 5). For each FFPE block, four consecutive sections were analyzed as technical replicates. (ii) DIA-MS analysis using two gradient lengths (45 min and 101 min), with comparison of data processing using a DDA-based spectral library and an in silico predicted library. **b**, The total number of precursors and protein groups stratified by gradient length and library. **c**, PCA illustrating clustering of samples for each library type and gradient length. PC1 explaining ~45–47% of the variability and PC2 accounting for ~16–18%. **d**, Hierarchical clustering dendrogram of quantified proteomes highlights consistent grouping by individual. **e**, Box plots showing the CV of all protein intensities across technical replicates from five individuals, for each condition. Box plots depict medians (center lines), IQR (boxes) and values within 1.5× the IQR (whiskers). Parts of panel **a** adapted from Servier Medical Art (https://smart.servier.com/) under a Creative Commons license CC BY 4.0. Source data

**Extended Data Fig. 3:**
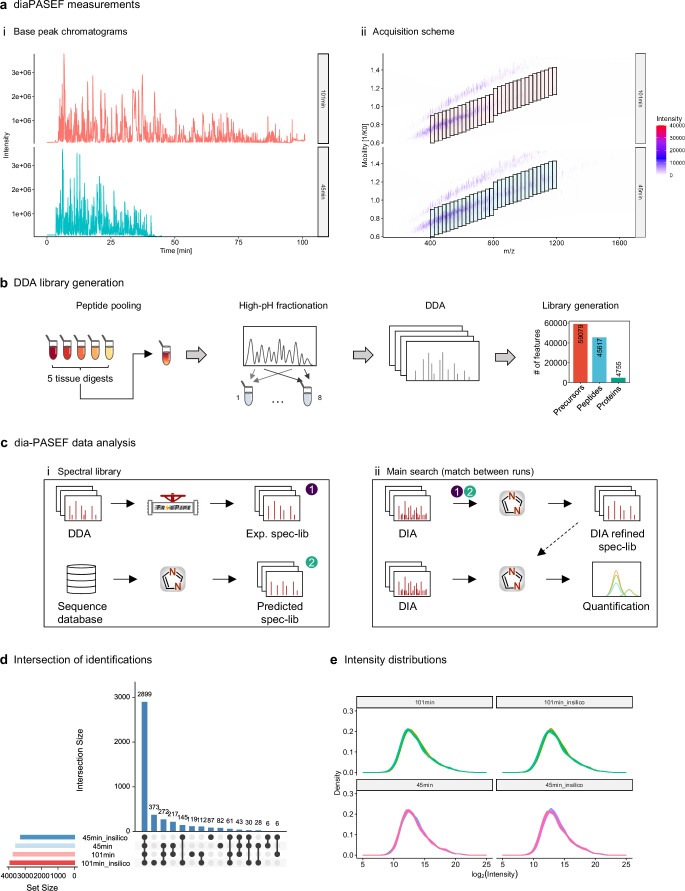
Workflow and analysis of dda- and dia-PASEF raw data. (**a**) i: Base peak chromatograms for the 101 min and 45 min gradients. ii: Acquisition scheme overlaid onto a scatterplot illustrating the m/z-ion mobility dimension. (**b**) Schematic workflow for DDA library generation. Peptides pooled from all patients were fractionated at high pH and subjected to dda-PASEF measurements to generate the experimental spectral library. (**c**) Schematic representation of dia-PASEF data analysis. i: Databases included the experimental spectral library (DDA library) and a predicted spectral library generated from the sequence database. ii: DIA data were analyzed using both libraries with match-between-runs (MBR) enabled for quantification. (**d**) Upset plot showing protein identifications across the two gradient durations and spectral library combinations. (**e**) Density plots of log2-transformed protein intensities. Source data

A significant outlook for proteomics profiling of cardiac FFPE samples is its potential to analyze tissues from retrospective clinical cohorts. Data-dependent acquisition (DDA) of proteomics data, as used in the previous section together with TMT multiplexing, has limitations in large-scale studies due to its reliance on sampling the most abundant peptides, often resulting in missing values and affecting data completeness across multiple samples. By contrast, data-independent acquisition (DIA) minimizes missing values, making it particularly suitable for large-scale studies. To evaluate the scalability of cardiac FFPE proteome profiling, we pursued single-shot DIA measurements on four separate FFPE sections from each of the five human hearts (Fig. 3a). The samples were analyzed on a trapped ion mobility spectrometry–time of flight (timsTOF HT) mass spectrometer in dia-PASEF (parallel accumulation–serial fragmentation) mode using two different analytical gradients: 45 min for a throughput of ~30 samples per day, and 101 min for a throughput of ~15 samples per day (Extended Data Fig. 3a). This in total generated data from 40 individual LC–MS runs. In the data processing, we evaluated two different approaches: using a sample-specific spectral library, generated from DDA measurements of a fractionated sample pool, or an in silico predicted (library-free) approach (Extended Data Fig. 3b,c).

Using the shorter 45 min gradient, we identified 33,051 peptide precursors mapping to 3,277 protein groups with an in silico, library-free analysis approach using deep learning-based predictions of retention time, ion mobility and intensities for scoring (Fig. 3b). With the longer 101 min gradient and the same data-processing strategy, we identified 41,357 precursors corresponding to 3,921 proteins, representing a 10% improvement in protein identifications. Evaluating the advantages of collecting a project-specific spectral library generated from offline fractionated sample pools, which comprised 59,079 precursors and 4,755 proteins, we observed the best performance with the 101 min gradient, identifying 3,729 proteins (36,863 precursors), whereas 3,577 proteins (34,596 precursors) were identified with the shorter gradient. The overlap in protein identifications across the two different LC gradients, analyzed with different libraries, is summarized in Extended Data Fig. 3d, showing that 2,899 proteins were consistently measured. Overall, the library-free analysis on the long gradient method yielded the most precursors and highest protein coverage.

PCA of the measured protein intensities (Extended Data Fig. 3e) showed that all four tested conditions could effectively distinguish proteome profiles across the five individuals (Fig. 3c). Despite variation in the analytical depth, both long and short gradients achieved equally distinct separation between samples. For both gradient lengths, data processing with either the project-specific library or the in silico library resulted in distinct clustering patterns of measured protein quantities, with a similar extent of variance explained. This indicates that we capture robust biological signals reflective of the respective individuals, independent of the analytical strategy. To further evaluate measurement consistency, we analyzed the coefficient of variation (CV) for individual protein intensities and performed hierarchical clustering on replicate samples (Fig. 3d,e). These analyses showed that technical variability was consistently low, with a median CV of 11%, indicating high reproducibility and quantitative precision across replicate measurements.

**Extended Data Fig. 4:**
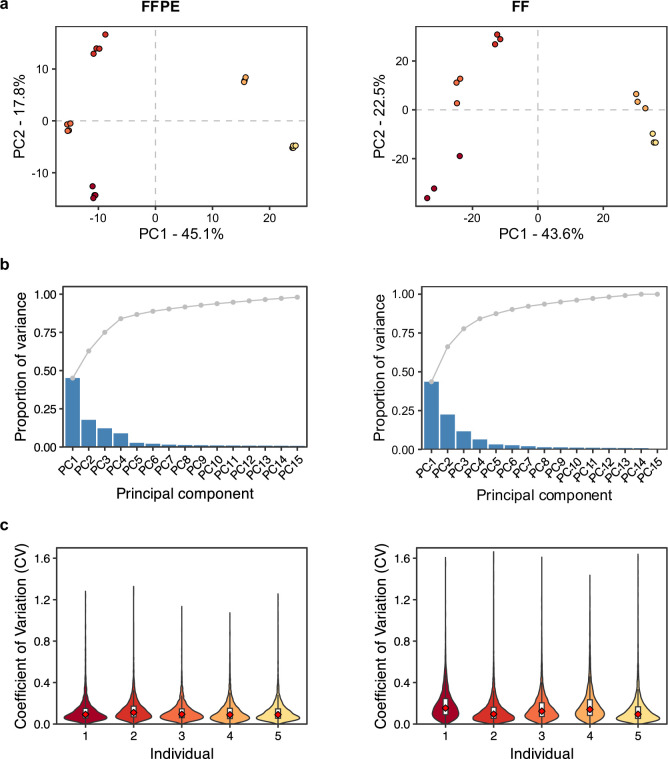
Comparison of proteome profiles from FFPE and matched fresh frozen heart tissue by label-free dia-PASEF. (**a**) Principal component analysis of protein intensities from five individuals, with 3–4 replicates per individual for FFPE and 3 replicates for FF samples. (**b**) Bar plots showing the proportion of variance explained by the top 15 principal components. (**c**) Violin plots showing the distribution of coefficients of variation (CV) across replicates for each individual. Embedded boxplots display the median (center line), interquartile range (box), and whiskers extending to 1.5× the IQR. The red diamond indicates the mean. Source data

To verify that the FFPE cardiac proteome profiles obtained using the DIA measurement strategy aligned with those of FF tissue, we conducted additional measurements on matched FF tissue samples from the same five individuals using the long DIA method and a library-free search strategy. PCA of protein intensities from FF samples closely mirrored the separation observed in FFPE datasets (Extended Data Fig. 4a). It is worth noting that the first two principal components accounted for over 60% of the total variance in both datasets and clearly separated the five individuals, highlighting that inter-individual proteomic differences are preserved regardless of tissue preservation method (Extended Data Fig. 4b). Quantitative precision, assessed by the CV, was also comparable, with a median CV of 12% across replicates, matching that of FFPE samples (Extended Data Fig. 4c).

We further stratified samples based on their separation along PC1 into two patient clusters and compared differential protein abundance between these groups within FF and FFPE datasets independently. Fold changes of significantly regulated proteins were strongly correlated (Pearson *R* = 0.86 for FFPE-significant, *R* = 0.84 for FF-significant and *R* = 0.92 for proteins significant in both), showing that abundance changes are reliably mirrored across preservation methods. A comparable analysis of FFPE samples measured by DIA and TMT also revealed high concordance (*R* = 0.84–0.93), confirming the robustness of proteomic signatures across workflows and tissue types.

We show that DIA measurements of cardiac proteome profiles provide a suitable alternative to TMT-based approaches, offering high analytical precision albeit with some trade-off in measurement depth. Our findings show that key proteomic signatures can be robustly captured using current deep-learning-based, library-free search strategies, supporting their utility alongside traditional library-dependent approaches, particularly in studies with limited sample availability. We further show that gradient length can be effectively shortened to reduce measurement time while preserving the biological signature of the sample cohort.

### Image-guided proteomics of the human sinoatrial node reveals molecular heterogeneity

**Fig. 4:**
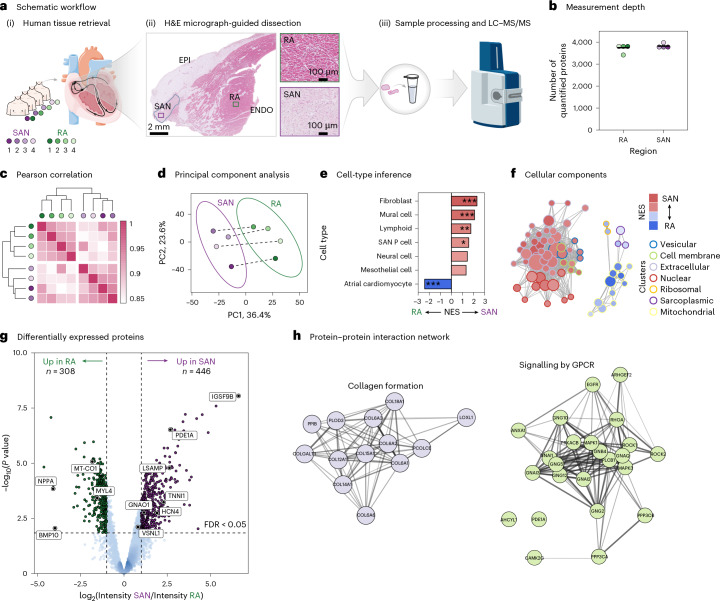
Proteomic profiling of the human SAN. **a**, (i) Overview of donor numbers (*N* = 4) and regions included in the study. (ii) Annotated H&E-stained image highlighting the SAN region (blue outline) used to guide tissue dissection. Magnified views show the pacemaker region (purple) and non-pacemaker region (green). ENDO, endocardium; EPI, epicardium. (iii) Proteomics data were generated from the SAN and RA regions on the timsTOF HT. **b**, Number of unique proteins quantified for each individual sample, with a horizontal line indicating the mean across samples. **c**, Clustered heatmap of pairwise Pearson correlation coefficients across sample from both regions. **d**, PCA of quantitative proteomics data. Ellipses represent 90% confidence regions for each group, and dashed lines connect samples originating from the same donor. **e**, Relative enrichment of cell-type markers among proteins with higher abundance in either the SAN or RA region. Enrichment scores were derived using GSEA. *P* values were adjusted for multiple testing using the Benjamini–Hochberg method. **f**, Functional enrichment of cellular components showing a clustered network based on term similarity of core enriched genes. **g**, Volcano plot representation of differentially expressed proteins between SAN and RA, assessed using empirical Bayes-moderated *t*-statistics with standard errors adjusted for within-donor correlation. Two-sided *P* values were Benjamini–Hochberg corrected. Dashed lines indicate significant up-regulation (log_2_FC > 1 and adjusted *P* < 0.05) or down-regulation (log_2_FC < −1 and adjusted *P* < 0.05). **h**, Protein–protein interaction network for selected pathways overrepresented among proteins with higher abundance in the SAN region. Adjusted *P* value codes: *P = 0.05; **P = 0.01; ***P = 0.001; exact *P* values are provided in the source data. Parts of panel **a** adapted from Servier Medical Art (https://smart.servier.com/) under a Creative Commons license CC BY 4.0. Source data

**Extended Data Fig. 5:**
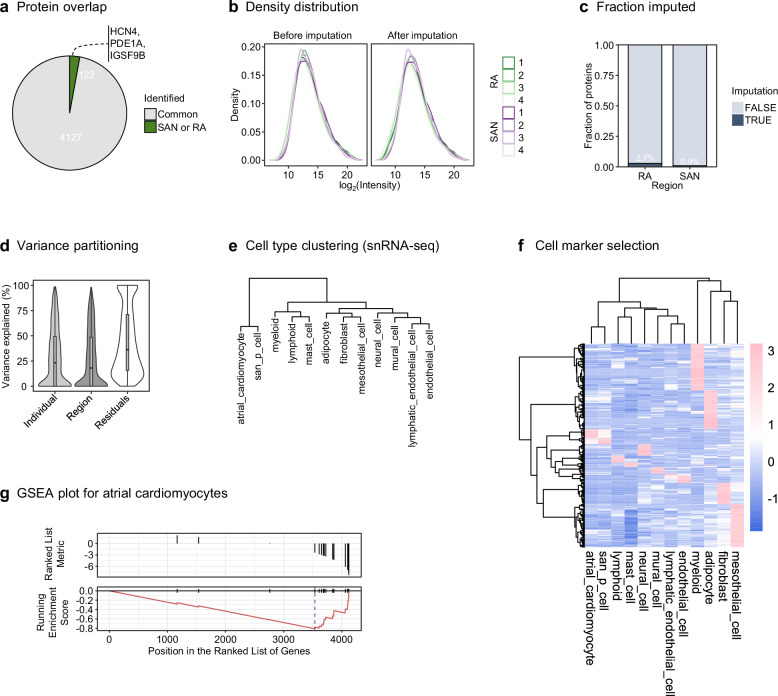
Quality control of proteomes from human sinoatrial node (SAN) and right atrium (RA) regions. (**a**) Overlap of proteins identified in the SAN and RA region. (**b**) Density plots of log2-transformed protein intensities before (left) and after (right) imputation of missing values. (**c**) Bar plot showing the fraction of proteins affected by imputation, stratified by region. (**d**) Variance partition plot showing the relative contributions of interindividual differences and regional differences to total proteomic variance. Boxplots indicating the median (center line), interquartile range (box), and whiskers extending to 1.5× the interquartile range (IQR). (**e**) Dendrogram of cell types based on average RNA expression profiles derived from single-nucleus RNA sequencing (snRNA-seq) data. (**f**) Hierarchical cluster heatmap of z-scored average expression levels of cell-type marker genes across clusters. (**g**) Representative Gene Set Enrichment Analysis (GSEA) plot showing enrichment of atrial cardiomyocyte markers toward the end of the ranked list (RA). Source data

The ability to resolve cardiac proteome profiles from FFPE cardiac specimens holds significant promise for investigating cardiac substructures and specific regions identified through histopathological analyses. To demonstrate the ability of our approach to investigate substructures that require histological image data for precise identification and targeted sample collection, we applied our FFPE-DIA workflow to archival human heart tissue containing the sinoatrial node (SAN). The SAN, with its unique molecular composition and complex morphology, presents significant challenges for study. We obtained archived heart tissue encompassing the SAN from four human hearts and conducted an unbiased proteomic analysis of the SAN compared to adjacent right atrial (RA) tissue (Fig. 4a). The SAN was localized using hematoxylin and eosin (H&E) stained sections, which guided the dissection of samples from both within the SAN and the adjacent RA myocardium. Following tissue homogenization and protein digestion, we performed label-free DIA-MS measurements, quantifying approximately 4,000 unique proteins in each sample using a library-free data-processing approach (Fig. 4b). Of the total 4,249 proteins identified in our cohort, 122 were exclusively found in either the SAN or the RA; among these were three known markers of the SAN: HCN4, PDE1A and IGSF9B (Extended Data Fig. 5a). The robust coverage of protein measurements across samples resulted in minimal missing values (Extended Data Fig. 5b,c). This, combined with high Pearson correlation coefficients of measured protein intensities (mean *R* = 0.91 within substructures) and clustering of samples by cardiac region (SAN versus RA) (Fig. 4c), provides an excellent foundation for quantitative comparisons. PCA of the protein intensities corroborates the hierarchical clustering results, with the primary source of variation in the data attributable to spatial differences, as evidenced by a distinct separation of SAN and RA samples along PC1 (Fig. 4d). The PC2 captures inter-individual differences as the second largest contributor to the overall variation in the dataset. Similar trends were observed by variance partitioning analysis (Extended Data Fig. 5d).

**Extended Data Fig. 6:**
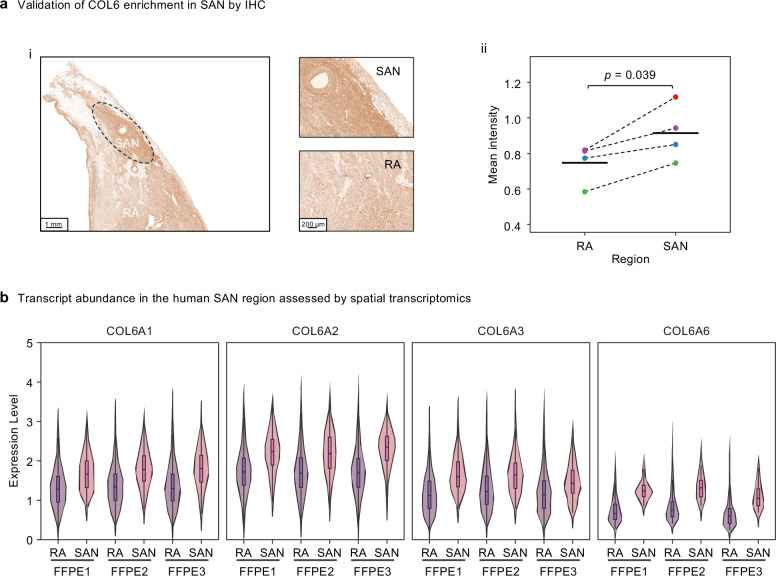
Validation of ECM and GPCR-related signatures in the human SAN. (**a**) Immunohistochemical validation of COL6 enrichment in the SAN. (i) Representative image of COL6 staining in FFPE heart section, with the SAN region outlined. Zoom-in panels on SAN and adjacent RA regions. (ii) Quantification of COL6 staining intensity (N=4 biological replicates) confirms significantly elevated expression in the SAN (two-sided paired t-test, p = 0.039). (**b**) Transcript abundance of COL6 family genes in the SAN versus RA assessed using a published spatial transcriptomics dataset^24^. Boxplots displaying the median (center line), interquartile range (box), and whiskers extending to 1.5× the interquartile range (IQR). Source data

To determine whether the proteomes measured from the SAN and the RA capture the well-established cellular differences between these regions, we leveraged recently published single-cell transcriptomics data of the human cardiac conduction system^24^. Using a deconvolution approach, we identified cell-type-specific markers and determined their relative abundances in the SAN compared to the RA (Extended Data Fig. 5e–g). The analysis revealed an enrichment of fibroblast and pacemaker cell markers within the SAN, while markers of atrial cardiomyocytes were predominantly expressed in the RA (Fig. 4e). This finding aligns with the understanding that pacemaker cells reside in a fibroblast-rich microenvironment^24–26^. The SAN was furthermore enriched in mural cells (smooth muscle cells and pericytes), lymphocytes and neural cells. Further supporting these results, functional enrichment analysis highlighted enrichment of specific cellular components in the SAN and RA. The SAN exhibited enrichment of extracellular matrix (ECM), plasma membrane and vesicular proteins, while the RA was characterized by an enrichment of sarcoplasmic and mitochondrial proteins (Fig. 4f). These functional enrichments are consistent with the cell-type deconvolution analysis, which underscores the prominence of fibroblasts and neuronal innervation in the SAN, alongside the more pronounced contractile phenotype of atrial cardiomyocytes compared to pacemaker cells. Next, we evaluated the dataset for differentially abundant proteins (Fig. 4g). The volcano plot shows a significant upregulation of SAN-specific markers (HCN4, PDE1A and TNNI1) in the SAN (Benjamini–Hochberg-adjusted *P* < 0.05, |logFC| > 1). VSNL1, a recently proposed SAN marker^27^, was significantly more abundant, although its fold change was below the set threshold. By contrast, established RA markers, including NPPA, BMP10 and MYL4, as well as mitochondrial proteins like MT-CO1 and MT-ATP6, were more abundant in the RA. Focusing on the SAN and evaluating protein–protein interaction networks among the significantly regulated proteins revealed two notable networks (Fig. 4h). One network highlighted the unique composition of the SAN’s ECM, which is enriched in non-fibrillar type VI collagen (COL6A1/2/3/6) and other non-traditional collagens, including types XII, XIV, XV and XVIII. Enrichment of type VI collagens in the SAN was validated by immunohistochemical staining (Extended Data Fig. 6a). Another significant network involved G protein-coupled receptor (GPCR) signaling, including GNAI1, GNAI2, GNAI3, GNG2, GNG5, GNB4, CAMK2G, PRKACB, GNAQ and GNAO1. Several of these proteins, particularly the inhibitory Gαi subunits (GNAI1/2/3), regulate adenylate cyclase activity and thereby cAMP levels, directly influencing heart rate. It is worth noting that GNAO1, which constitutes the G protein subunit alpha O1, was recently proposed as a SAN-specific marker in mice^28^. Our findings support that GNAO1 is also a protein marker of the human SAN. Transcript levels corresponding to all proteins depicted in the protein interaction networks in Fig. 4h were found to be significantly enriched in the SAN region compared to adjacent RA myocardium, as validated using an independent, publicly available spatial transcriptomic dataset of the human sinus node (Extended Data Fig. 6b and Supplementary Tables 4 and 5)^24^.

**Fig. 5:**
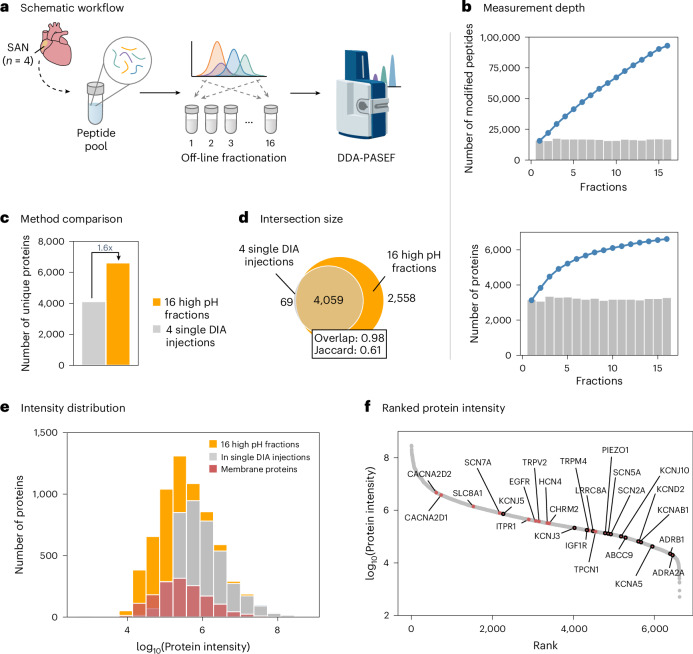
Expanded proteome coverage of the human SAN proteome by off-line high pH fractionation. **a**, Schematic workflow illustrating the reprocessing of SAN samples from four individuals. Peptides from individual preparations were pooled for off-line high-pH fractionation before dda-PASEF acquisition. **b**, Cumulative number of modified peptides (top) and proteins (bottom) identified across 16 fractions, with individual bars representing per-fraction identifications. **c**, Comparison of total unique protein identifications between the fractionated approach and single-shot DIA measurements of the four individual SAN preparations. **d**, Overlap analysis of proteins identified by both methods. **e**, Protein intensity distribution from the fractionated dataset, highlighting proteins also found in single-injection DIA and membrane-annotated proteins. **f**, Ranked protein abundance from the deep SAN proteome, with uniquely identified ion channels/receptors highlighted by a black outline. Source data

Given that GPCR signaling networks and distinct ion channel expression profiles are central to the molecular identity of the SAN, we sought to extend our previous spatial proteomic analysis by increasing measurement depth using the same SAN tissue samples. To this end, peptide material from the four SAN samples profiled in Fig. 4 was pooled to enable off-line high-pH reversed-phase fractionation, followed by DDA-PASEF acquisitions across 16 peptide fractions (Fig. 5a). This approach substantially expanded the detectable SAN proteome, yielding over 6,600 uniquely identified proteins—1.6-fold more than identified in the single-shot DIA measurements of the individual SAN samples (Fig. 5b–d). It is worth noting that there was a 98% overlap with the DIA dataset, showing strong concordance between approaches. The increased depth improved coverage of low-abundance and membrane-associated proteins, including challenging targets such as β1- and α2A-adrenergic receptors (Fig. 5e,f). These results highlight the value of deep proteome profiling in spatially resolved tissues and further characterize the human SAN proteome at high resolution.

Collectively, these results demonstrate that image-guided macro-dissection of cardiac FFPE samples enables the capture of distinct proteomic signatures from specialized regions. By focusing on the SAN, integrating histology and high-sensitivity proteomics, we provide a detailed protein map of the human SAN, offering complementary insights to its known cellular heterogeneity.

### Proof-of-principle proteomic profiling of human myocardial biopsies from a retrospective patient cohort outlines the distinct signature of patients with ACM

**Fig. 6:**
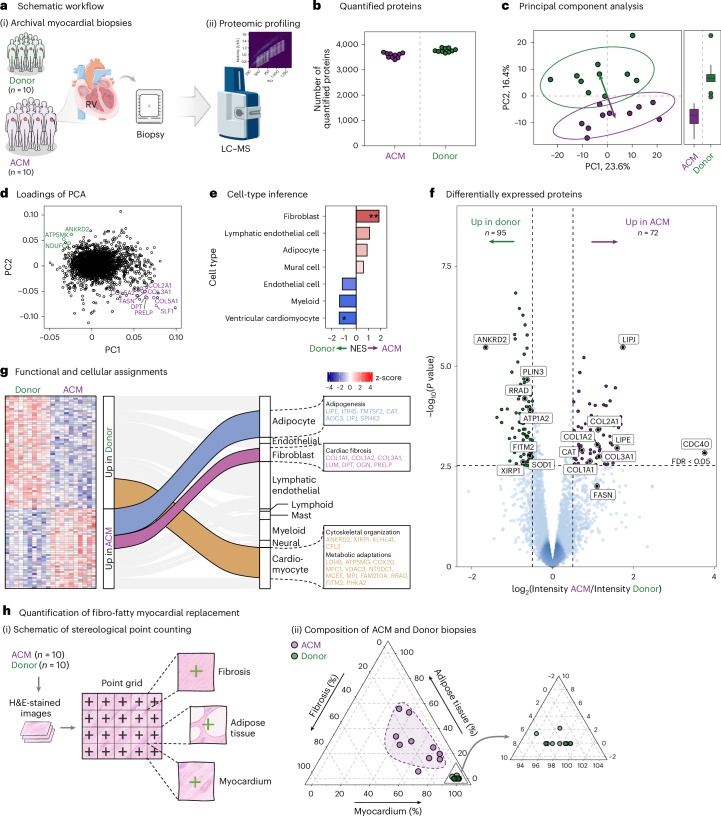
Proteomic profiling of 20 human myocardial biopsies from patients with ACM and donor hearts. **a**, (i) Overview of the patient cohort included in the study. (ii) Deep proteomics data were generated from patients with ACM (*N* = 10) and donor hearts (*N* = 10) on the timsTOF HT in dia-PASEF mode. **b**, Number of unique proteins quantified from each individual biopsy. **c**, Dimensionality reduction by PCA. Left: Representation of samples along the first two PCs; 90% confidence intervals for ACM and donor groups are depicted as ellipses. Arrows point at the centroid of the respective clusters. Right: Box plots showing separation of samples along PC2. Boxes indicate the IQR, center lines mark medians, and whiskers extend to 1.5× the IQR. **d**, Protein loadings along PC1 and PC2. Proteins aligning with the direction of the vectors separating ACM and donor groups are highlighted. **e**, Bar graph representation of cell-type marker enrichment results. NESs are shown; *P* values were obtained by permutation testing and adjusted for multiple testing using the Benjamini–Hochberg method. **f**, Volcano plot representation of differentially expressed proteins between ACM and donor tissues, assessed using empirical Bayes-moderated *t*-statistics. Two-sided *P* values were Benjamini–Hochberg adjusted. Dashed lines indicating significant up-regulation (log_2_FC > 0.5 and Benjamini–Hochberg-adjusted *P* < 0.05) or down-regulation (log_2_FC < −0.5 and Benjamini–Hochberg-adjusted *P* < 0.05). **g**, Heat map of *z*-scored protein intensities for differentially expressed proteins. The alluvial plot links upregulated (higher abundance in ACM) and downregulated (lower abundance in ACM) proteins to their likely cell-type origins. **h**, Quantification of fibro-fatty myocardial replacement in ACM hearts. (i) Schematic illustrating the stereological point counting method used to quantify tissue composition from H&E-stained sections by categorizing each grid point as fibrosis, adipose tissue or myocardium. (ii) Ternary plot showing the relative tissue compositions in biopsies from ACM (purple) and donor (green) hearts. A zoom-in panel highlights the donor cluster for improved visibility. Dashed line indicates the 75% confidence interval. Adjusted *P* value codes: *P = 0.05; **P = 0.01; exact *P* values are provided in the source data. Parts of panel **a** adapted from Servier Medical Art (https://smart.servier.com/) under a Creative Commons license CC BY 4.0. Source data

**Extended Data Fig. 7:**
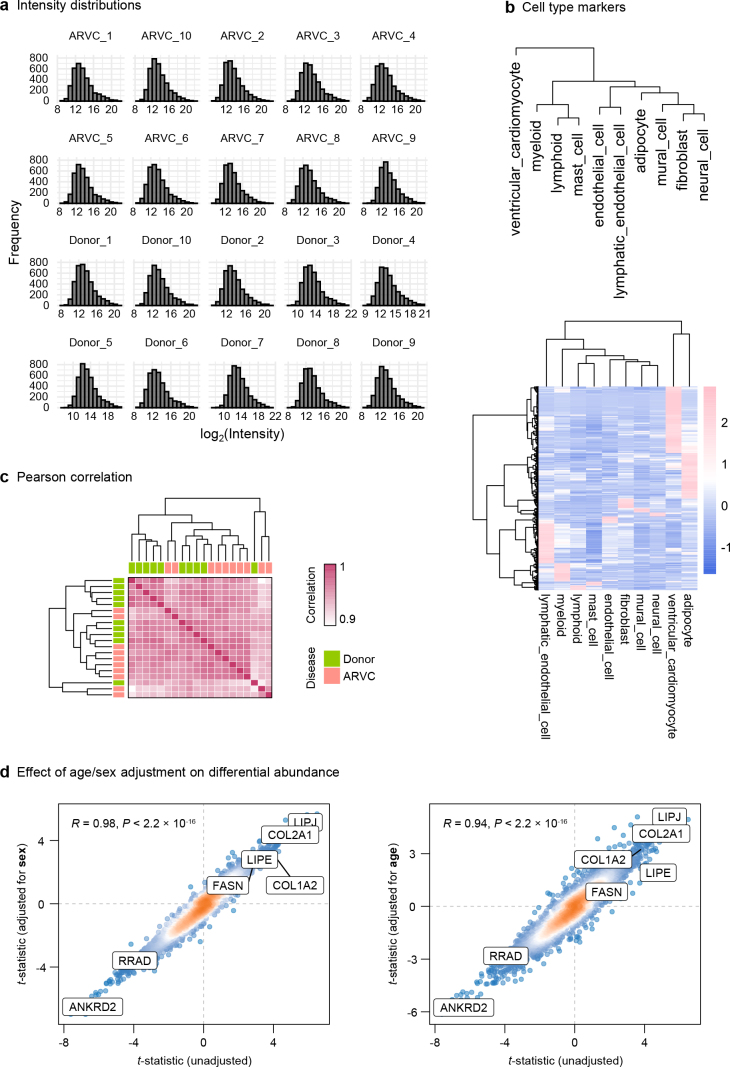
Proteomic quality control and covariate adjustment in arrhythmogenic cardiomyopathy (ACM) and donor heart samples. (**a**) Histogram of log2-transformed protein intensities across 10 ACM and 10 donor samples. (**b**) Clustered heatmap of pairwise Pearson correlation coefficients between all samples. (**c**) Cell type marker detection. Upper panel: Hierarchical cluster heatmap of z-scored average RNA expression per cell type. Lower panel: Hierarchical cluster heatmap of z-scored average expression levels of cell-type marker genes across clusters. (**d**) Effect of covariate adjustment on differential protein abundance comparisons between ACM and donor samples. Scatter plots compare t-statistics from unadjusted models (x-axis) to those from models adjusted for sex (left) or age (right) (y-axis). Pearson correlation coefficients (R) quantify the consistency between models. P-values are two-sided. Source data

We next investigated the feasibility of using our established workflow to profile cardiac disease states in myocardial biopsies from archival FFPE tissue collected for diagnostic purposes in a clinical setting. Our retrospective proof-of-concept study included endomyocardial biopsies from ten patients diagnosed with ACM and comparable biopsies from ten donor hearts used for transplantation as controls (Fig. 6a(i)). Following our sample processing workflow and data acquisition via single-shot DIA analysis (Fig. 6a(ii)), we identified a total of 4,484 unique proteins across all samples. Of these, 4,055 were retained for quantitative analysis based on data completeness criteria across the ACM and donor cohorts (Fig. 6b and Extended Data Fig. 7a,c). PCA of the measured proteins revealed a distinct separation between ACM and donor groups along the first two principal components, effectively classifying samples based on disease state (Fig. 6c). This indicates that the acquired proteome profiles capture the signature of the disease state.

Inspection of the PCA loadings, representing the major protein contributors to principal components, highlighted proteins linked to the histological hallmarks of ACM, such as COL3A1, COL5A1 and PRELP for fibrosis and FASN for fatty infiltrates (Fig. 6d). We integrated our proteome data with published single-nucleus transcriptomics data from human hearts to identify cell-type specific markers (Extended Data Fig. 7b). Analysis of these markers showed a significant enrichment of fibroblast markers in ACM biopsies, alongside a notable trend for adipocyte markers (Fig. 6e). By contrast, markers for ventricular cardiomyocytes were significantly more abundant in Donor hearts compared to those of patients with ACM.

Further analysis of differentially expressed proteins between ACM and Donor hearts identified 167 proteins with significantly altered abundance (Benjamini–Hochberg-adjusted *P* < 0.05, |logFC| > 0.5). Among these, 72 proteins were more abundant and 95 were less abundant in ACM hearts compared to Donor hearts (Fig. 6f). ACM hearts showed decreased levels of cytoskeletal proteins ANKRD2 and XIRP1 and increased levels of multiple fibrosis-related collagens, including type I and II collagens, as well as TGF-beta modulators DPT and OGN (Fig. 6f). In addition, we observed elevated abundances of catalase, which is important for protection against oxidative damage^29,30^, and hormone sensitive lipase, indicating increased triglyceride metabolism in biopsies from patients with ACM.

**Extended Data Fig. 8:**
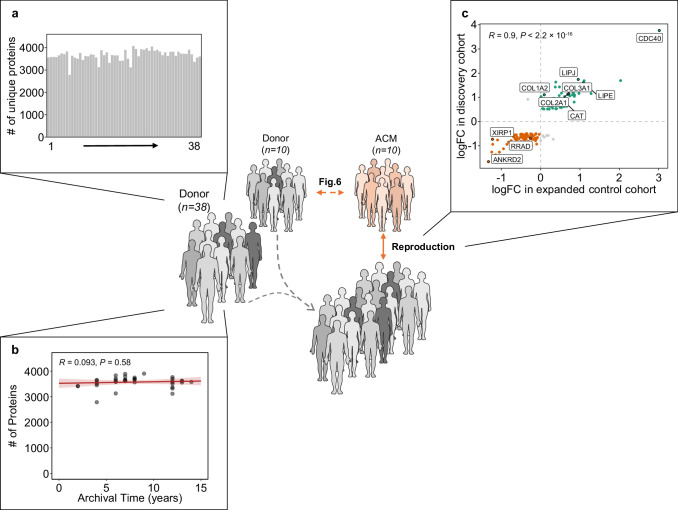
Reproducibility of differential protein abundance in ACM cases versus an expanded donor cohort. Overview of study design: Proteomic profiling of an expanded set of donor hearts (n = 38) was performed to evaluate the reproducibility of findings from the initial ACM versus donor comparison (Fig. 6). (**a**) Number of uniquely identified proteins across the 38 donor samples. (**b**) Correlation between the number of identified proteins per sample and the storage time of the corresponding FFPE blocks. A linear regression line is shown, with the shaded area representing the 95% confidence interval. The Pearson correlation coefficient (R) and its associated two-sided p-value are displayed. (**c**) Comparison of log2 fold changes between the discovery cohort (ACM vs. 10 donors; y-axis) and the reproduction cohort (ACM vs. 48 donors; x-axis). Selected consistently upregulated proteins are annotated. The Pearson correlation coefficient (P = 4.3 × 10^−31^, two-sided) is displayed. Source data

To ensure that demographic differences, particularly age and sex, did not confound the observed proteomic differences between ACM and donor hearts, we evaluated their impact by fitting models with and without these covariates. Comparison of the resulting *t*-statistics across all proteins revealed strong concordance (Pearson *R* > 0.94; Extended Data Fig. 7d), indicating minimal influence on the overall differential abundance patterns. To further substantiate that the protein profiles distinguishing the ACM from donor hearts are not confounded by these factors, we acquired proteomic profiles from 38 additional donor heart biopsies (Extended Data Fig. 8a). A combined analysis using our expanded, better-matched control cohort yielded effect sizes that correlated strongly with those from the original analysis (Extended Data Fig. 8c), reinforcing that the observed proteomic signature likely reflects disease-specific alterations. We also assessed the impact of FFPE block storage time on proteome quality within this expanded cohort and found no significant association with the number of identified proteins (*R* = 0.093; Extended Data Fig. 8b).

To elucidate the cellular origins of the protein differences between ACM and donor hearts depicted in the volcano plot, we used single-nucleus transcriptomics data from human hearts. For all proteins with significant differences between ACM and donor hearts, we identified the primary cardiac cell type expressing each protein (Fig. 6g). This systematic reconstruction of cell type–protein associations effectively restores information lost in bulk proteomic analyses and provides links between cell-type enrichment and significantly regulated proteins. Our analysis shows that significantly regulated cardiomyocyte proteins are predominantly downregulated in ACM hearts compared to donor hearts, while significantly regulated adipocyte and fibroblast proteins are predominantly upregulated in ACM hearts (Fig. 6g). Figure 6g highlights key regulatory changes within cardiomyocytes, fibroblasts and adipocytes that distinguish the proteome profile of ACM hearts from those of donor hearts.

To verify the observed increases in adipocytes and fibrosis in ACM hearts relative to donor hearts, we performed quantitative histological evaluations of H&E-stained endomyocardial biopsy sections from the 10 patients with ACM and 10 donor controls. Using a stereological point counting approach, each tissue section was systematically scored for myocardium, fibrosis and adipose tissue. This analysis confirmed a higher proportion of adipose and fibrotic tissue in ACM hearts compared to controls (Fig. 6h).

These results show the capability of our approach to uncover molecular hallmarks of disease and identify specific protein remodeling characteristic of cardiac disease states by using retrospectively collected clinical biopsies.

## Discussion

The application of FFPE tissue in omics research has become increasingly widespread, particularly in oncology; however, its potential for proteomic analysis of cardiac tissues remains underexplored. Previous studies investigating FFPE human heart tissue demonstrated feasibility but faced significant limitations. These included the identification of only a few hundred^14^ to low-thousand proteins^15,31^, reliance on xylene-based deparaffinization steps, non-parallelized sample processing workflows and the need for long chromatography gradients, all of which constrained scalability and throughput. At the same time, uncovering meaningful insights into the multifactorial nature of cardiovascular diseases—which result from a complex interplay of genetic variants, environmental factors and comorbidities—necessitates overcoming existing barriers to tissue acquisition by transitioning from the reliance on FF tissue to FFPE tissue while achieving comparable proteome coverage. This benchmark, particularly for human cardiac tissue, had not been reached until now.

Simultaneously, increasing the widespread use of cardiac FFPE tissue facilitates the study of biopsies collected from living patients during clinical diagnosis, offering unique biological advantages over FF samples typically obtained post-mortem or at explant. This access to earlier or intermediate disease stages is critical for investigating dynamic pathological processes, where molecular changes are often subtle and spatially localized before extensive remodeling occurs.

In this Article, we demonstrated how FFPE cardiac samples can be leveraged to uncover key molecular pathways in heart disease and resolve spatial substructures, bridging traditional histopathology with proteomics. We show that this approach is applicable to a wide range of clinical and research settings in the field of cardiology, where FFPE tissues are more readily available than their FF counterparts and can be archived over long periods.

We highlight how recent technological improvements, including efficient removal of detergents and hydrophobic contaminants, plate-based sample processing, next-generation mass spectrometry and advanced computational tools, allow us to now pursue FFPE-based proteomics as a viable alternative to the current gold standard of FF tissue studies. Our scalable 96-well workflow quantifies expressed proteins in an untargeted manner from minimal archival material, accommodating the limited tissue availability from punch biopsies or even macrodissected tissue. This method routinely achieves a depth of ~4,000 unique proteins, enabling the comprehensive characterization of (sub-)proteomes within heart tissues. These insights offer a robust foundation for identifying novel therapeutic targets, diagnostic markers and patient stratification strategies.

Addressing concerns regarding formalin-induced crosslinking, our experimental design effectively accounted for these effects, leaving minimal attributable variance at the protein level—a finding consistent with observations in other fields^32,33^. We further demonstrate that proteomic signatures from matched FFPE and FF tissues derived from the same hearts are highly reproducible, supporting the comparability of these tissue types. However, due to the technical challenges in acquiring such uniquely matched human heart tissue samples, these comparisons were restricted to our benchmark set and could not be extended to the biological case studies presented. The validity and biological relevance of FF versus FFPE proteomes have been addressed in murine models^34^ and oncology samples^35^, providing a foundation for future studies to validate FFPE proteomes in well-matched human heart disease cohorts.

Our FFPE workflow supports extensive fractionation with isobaric labeling for deep proteome coverage. While small-scale TMT set-ups, such as the single-batch design used here, enable highly complete datasets and precise comparisons, scaling to multi-batch experiments introduces challenges including missing value inflation and batch effects^36^, which require careful normalization and integration. These issues impact both scalability and data consistency, highlighting the need for computational tools that specifically address the unique missing-value patterns and hierarchical data structures resulting from sample nesting within TMT plexes. Ultimately, these limitations motivated our parallel evaluation of label-free DIA, which benefits from deterministic sampling, resulting in high reproducibility and reduced missingness across large cohorts. In particular, single-shot dia-PASEF measurements offer an efficient balance between depth and scalability, enabling robust quantification. We demonstrate that traditional prerequisites such as sample pooling and offline fractionation for DDA acquisitions and library generation are no longer essential, with library-free approaches now delivering comparable depth and precision^37^.

Our case studies highlight the utility of this approach in uncovering key proteomic hallmarks in anatomical regions like the human sinus node and disease states such as ACM. The SAN’s intricate, crescent-like structure beneath the epicardial surface poses significant challenges for study. Using histology-guided dissection, we obtained detailed proteomic profiles of the SAN, providing a molecular characterization that reflects its unique composition. These profiles revealed the SAN’s extensive connective tissue (collagens and fibroblasts) and a distinctive pacemaker cell profile with fewer mitochondria and reduced sarcoplasmic reticulum/myofilaments compared to atrial cardiomyocytes. In addition, neurohormonal regulation of heart rate was evident in enriched GPCR signaling proteins, including established SAN markers such as PDE1A, GNAI1/2/3, CAMK2G and PRKACB, along with recently suggested markers like GNAO1, underscoring the power of spatial proteomics in resolving subregional cardiac biology. As SAN profiling relied on autopsy-derived tissue, post-mortem interval represents a potential confounder. A key strength of our study is the paired design, comparing SAN and adjacent RA from the same individual, which effectively controls for inter-individual sources of variation, including post-mortem interval. For studies lacking such internal controls, this factor should be carefully considered during both experimental design and downstream interpretation, as recently demonstrated^38^.

In the second case study, we evaluated the workflow’s utility in disease profiling by analyzing archival myocardial biopsies from patients with ACM alongside matched control samples from heart transplants. ACM, histologically characterized by fibro-fatty myocardial infiltration, provides a unique model to assess whether proteomic profiles recapitulate pathological features. Deep proteomic profiling of low-input FFPE myocardial biopsies revealed molecular hallmarks of ACM, including proteins associated with fibrosis and metabolic dysregulation, validating the workflow’s applicability to disease contexts. As with other retrospective FFPE studies, we acknowledge that archival time may introduce confounding effects due to potential protein degradation or chemical modifications over prolonged storage. However, previous investigations have shown that FFPE tissue blocks stored for 10–15 years retain sufficient integrity for deep and reproducible protein quantification^19,39^. Consistent with these findings, we observed no data quality issues attributable to block age in our dataset. Nonetheless, we highlight archival time as a relevant factor for consideration in retrospective proteomic studies, especially when comparing tissues stored over disparate time frames.

With further advancements in proteomics technologies and potential integration with approaches like laser capture microdissection, we anticipate studying cardiac tissue at unprecedented depth and spatial resolution, ultimately revealing molecular hallmarks that have previously been inaccessible. This progress opens the door to more comprehensive and robust cardiac studies, enabling the discovery of novel biomarkers and therapeutic targets.

## Methods

### Ethical approval and human heart samples

Proteomic studies of human heart samples were carried out in agreement with Danish Law (Medical Research Involving Human Subjects Act) and institutional guidelines, with ethical approval from The Regional Committee on Research Ethics (H-22012592) or in accordance with ethical standards for method development studies in Denmark. Dispensation from informed consent was granted by the Ethics Committee (H-22012592). The data presentation is in accordance with the European Union General Data Protection Regulation (EU GDPR) article 6(1) regarding public interest and 9(2)(j) regarding processing of special categories of personal data. Information on all human samples is provided in Supplementary Tables 1–3.

#### Left ventricular tissue from five human hearts

Post-mortem human heart samples were collected from five individuals. Samples from the left ventricular myocardium were collected as routine sampling following international guidelines during clinical autopsies performed at the Department of Pathology, Copenhagen University Hospital. Each heart sample was split into two parts: one half was snap frozen in liquid nitrogen, while the other half was immersed in 10% neutral buffered formalin for 6 h, dehydrated and paraffin-embedded using routine methods.

#### Archived human heart FFPE samples containing the sinus node

Archived human heart samples were retrieved from four individuals. Samples were collected post-mortem, where the samples from the conduction system were obtained as routine sampling following international guidelines during clinical autopsies performed at the Department of Pathology, Copenhagen University Hospital. The heart samples were immersed in 10% neutral buffered formalin for 6 h, dehydrated and paraffin-embedded using routine methods. Microsections were cut at a thickness of 4 μm, deparaffinized and stained with hematoxylin using routine methods. The stained microsections were imaged with a ×40 objective lens (Nanozoomer S360, Hamamatsu, Japan). The sinus node region was identified from the hematoxylin staining.

#### Archived human heart biopsies from retrospective clinical cohort

Endomyocardial biopsies collected for clinical diagnosis were retrieved from a repository of FFPE-preserved samples archived under standardized conditions before analysis, with a maximum storage duration of 15 years. Biopsies were obtained from the apical septum of the right ventricle using X-ray-guided collection, performed by advancing a bioptome through a catheter placed via the right internal jugular vein. After collection, the biopsies were immersed in 10% neutral buffered formalin for 6 h, dehydrated and paraffin embedded using routine methods. FFPE blocks were obtained from 10 patients with a diagnosis of ACM, as well as a total of 48 biopsies collected from recipients of a heart transplant.

### Proteomic sample preparation

#### Protein extraction from FFPE tissue

FFPE blocks were sectioned on a microtome into slices of 50–100 µm thickness. Excess paraffin was removed using a sterile scalpel, and tissue was diced into smaller pieces. The resulting pieces were transferred to a 96-well plate containing 100 µl of lysis buffer (5% SDS, 100 mM Tris, pH 8.5). Samples were allowed to rehydrate overnight followed by a single round of sonication using a PIXUL multi-sample sonicator for 30 min (50 N pulse, 1 kHz, 20 Hz burst rate). The plate was briefly centrifuged before being placed on a heat block for 1 h at 80 °C. After another spin-down step, sonication was repeated for an additional 30 min or until completely homogenized. Protein amount, as determined by BCA assay, was matched across all samples.

#### Protein extraction from FF tissue

FF tissue samples were stored at −80 °C and kept on dry ice until homogenization. Tissue was collected using a sterile scalpel and transferred either to a 96-well plate for processing as described above or to a Precellys tube containing four 1.4 mm zirconium beads (FF reference workflow^11^). Lysis buffer (50 mM Tris, pH 8.5, 5 mM EDTA, 150 mM NaCl, 10 mM KCl, 1% Triton, protease inhibitor cocktail) was added at a ratio of 200 µl per 20 mg of tissue. Homogenization was performed on a Precellys homogenizer for 25 s at 5,500 r.p.m. After homogenization, samples were incubated at 4 °C for 2 h under continuous head-over-head rotation to solubilize protein. Following incubation, samples were centrifuged at 10,000 × *g* for 10 min at 4 °C to pellet any debris. Protein concentration in the supernatant was then quantified by BCA assay, and concentrations were adjusted across all samples.

#### S-trap sample processing

A maximum of 100 µg of protein per sample was diluted to a final volume of 46 µl using SDS lysis buffer. Proteins were reduced with 5 mM tris(2-carboxyethyl) phosphine at 55 °C for 15 min and alkylated with 10 mM chloroacetamide at room temperature for 30 min. Samples were acidified with aqueous phosphoric acid and diluted 6-fold with binding buffer (100 mM triethylammonium bicarbonate (TEAB), 90% methanol). The resulting solutions were loaded onto S-trap mini spin column or S-trap 96-well digestion plates and washed with binding buffer once, followed by three washes with a 50% methanol/50% chloroform solution to remove paraffin and other hydrophobic contaminants. Additional washing with binding buffer was performed twice. Proteins were digested overnight at 37 °C using a combination of trypsin and Lys-C at ratios of 1:10 and 1:50 (*w*/*w*) in 50 mM TEAB. Tryptic peptides were eluted from the column/plate, first with 50 mM TEAB, followed by 0.2% formic acid and finally 50% acetonitrile (ACN). The eluates were pooled and acidified with 1% trifluoroacetic acid (TFA), and ACN was removed via vacuum centrifugation at 60 °C. Peptides were desalted using C18 resin and subsequently dried under vacuum.

#### In-solution digestion of FF tissue

As an alternative to S-trap processing, proteins were processed as recently described^11^. Briefly, protein samples were precipitated by adding four volumes of ice-cold acetone and incubated at −20 °C for 30 min. Precipitated proteins were pelleted by centrifugation at 2,000 × *g* for 2 min at 4 °C. After carefully discarding the supernatant, the protein pellets were air-dried for 15 min and subsequently resuspended in 6 M guanidine hydrochloride (Gdn-HCl) in 50 mM Tris buffer (pH 8.5). Reduction and alkylation were performed by adding tris(2-carboxyethyl) phosphine (5 mM) and chloroacetamide (10 mM) to the samples, followed by heating at 90 °C for 5 min with shaking at 1,000 r.p.m. The concentration of Gdn-HCl was then diluted to 2 M using 50 mM Tris buffer. Lys-C was added at a ratio of 1:50 (*w*/*w*) for pre-digestion, which was carried out for 1 h at 37 °C. After further diluting the Gdn-HCl concentration to 0.5 M, overnight digestion was performed with trypsin (1:100, *w*/*w*) at 37 °C, 700 r.p.m. The resulting digests were acidified with 1% TFA and centrifuged at 14,000 × *g* for 10 min, and the supernatant containing the peptides was desalted on SepPak C18 columns. The eluate was dried under vacuum.

#### TMTpro labeling

Peptide labeling with tandem mass tag (TMTpro) reagents was performed as previously described^40^. Dried peptides were resuspended in 50 mM HEPES buffer (pH 8.5) at a final concentration of 1 µg µl^−1^ and incubated with TMTpro reagents (final ACN concentration of 20%, peptide-to-TMT ratio of 1:5). The labeling reaction was carried out for 1 h at 1,000 r.p.m. at room temperature. After labeling efficiency check (>98%), the reaction was quenched by adding 1% hydroxylamine, and incubation continued for an additional 15 min at room temperature with 1,000 r.p.m. agitation. The samples were then combined and acidified with 1% TFA, and ACN was removed by vacuum centrifugation. Peptides were desalted using self-packed C18 StageTips before fractionation.

#### High pH fractionation

Dried peptides were resuspended in 10 mM TEAB and adjusted to a concentration of 1 µg µl^−1^ (20 µg). Fractionation was performed using a 100 min multi-step gradient on an Easy-nanoLC system, coupled to an Opentrons OT-2 for automated sample concatenation, as recently described^40^. The mobile phase consisted of buffer A (10 mM TEAB) and buffer B (10 mM TEAB, 80% ACN). The resulting fractions were dried under vacuum and resuspended at a concentration of 0.1 µg µl^−1^ in 0.1% TFA and 2% ACN.

### LC–MS/MS measurements of TMT labelled samples

LC–MS/MS measurements were performed on a Vanquish Neo system coupled to an Orbitrap Eclipse Tribrid mass spectrometer (Thermo Fisher Scientific). Samples were separated on a 200 cm µPAC column (315 µm bed width) at a flow rate of 300 nl min^−1^ over a 165 min gradient. The mobile phase gradient was generated by a binary solvent system, consisting of 0.1% formic acid in water (buffer A) and 0.1% formic acid in 80% ACN (buffer B). The gradient profile was as follows: 5% buffer B for the first minute, followed by a linear increase from 5% to 30% B over 105 min (1–110 min), from 30% to 50% buffer B over the next 5 min (110–115 min), from 50% to 97.5% buffer B over the subsequent 5 min (115–120 min), held at 97.5% buffer B for 5 min (120–125 min), followed by a linear decrease from 97.5% to 1% buffer B over 5 min (125–130 min), and finally held at 1% buffer B for the remaining 35 min (130–165 min). Column effluent was ionized using a nanospray ionization source with a spray voltage of 2.2 kV and an ion transfer tube temperature of 275 °C. Precursor ion scans were acquired in the Orbitrap at a resolution of 120,000 with a scan range of 400–1,400 *m*/*z*, a maximum injection time of 50 ms and a normalized automatic gain control target of 100%. For fragment ion scans, peptides with charge states of 2–6 were selected in DDA mode with a dynamic exclusion duration of 45 s and a mass tolerance of 10 ppm. A minimum intensity threshold of 50,000 and a maximum intensity threshold of 1 × 10^20^ were applied. The quadrupole isolation width was set to 0.7 Da, and fragmentation was performed by higher-energy collisional dissociation at a collision energy of 35%. Fragment ions were analyzed in the Orbitrap at a resolution of 50,000, with a maximum injection time of 120 ms and a normalized automatic gain control target of 200%.

### Raw data processing (TMT)

Thermo raw files were converted to mzML format using MSConvert and subsequently processed using FragPipe (v20) utilizing the ‘TMT16’ workflow. MS/MS spectra were searched against a human protein sequence database provided as a FASTA file (UP000005640, downloaded on 22 September 2023), which included reviewed protein sequences, common contaminants and decoy sequences. The MSFragger^41^ (v3.8) search parameters specified carbamidomethylation of cysteine and TMTpro labeling of lysine as fixed modifications. Variable modifications included oxidation of methionine, acetylation at the protein N-terminus and TMTpro labeling of the peptide N-terminus, allowing up to three variable modifications per peptide. PSMs were rescored using Percolator^42^ (v3.06.0), and protein inference was performed with ProteinProphet (v5.0.0). Results were filtered with Philosopher^43^ (v5.0.0) to a 1% false discovery rate (FDR) at both the precursor and protein levels. For isobaric quantification, quantitative reports were generated with TMT-Integrator (v4.0.5) using default PSM filtering criteria, with the following modifications: a precursor ion purity threshold of at least 0.75 and unique mapping of the amino acid sequence to a single protein (unique peptides). Manufacturer-provided isotopic correction factors were not applied to the data. To convert ratios to abundances, summed TMT reporter ion intensities were used. The results were aggregated at the protein level, log_2_ transformed and median normalized for downstream analysis in RStudio (2024.04.2).

### Bioinformatic analysis of TMT-multiplexed data

The psm.tsv file containing FDR-filtered search results was stripped of contaminants and used to determine the number of identified features. Subsequent analyses were based on isobaric quantification data at the protein level, generated by TMT-Integrator. Only proteins with complete observations across channels 126–133C were retained. Clustering analysis was performed in R (v4.4.1) using the stats package. Pairwise distances between samples were calculated using Euclidean distance (‘dist’ function), followed by hierarchical clustering using Ward’s D2 method (‘hclust’ function). Pearson correlation coefficients were computed using the ‘cor’ function and visualized with the ‘pheatmap’ function from the pheatmap package (v1.0.12), applying Euclidean distance for clustering. PCA was conducted with the ‘prcomp’ function.

GSEA was performed on the ranked loadings of PC1 using the ‘gseGO’ function from the clusterProfiler package^44^ (v4.12.1), focusing on cellular components as subontologies with a minimum gene set size of 100. *P* values were corrected using the Benjamini–Hochberg method, and a significance cut-off of 0.05 was applied. Significantly enriched gene sets associated with positive loadings on PC1 (normalized enrichment scores (NES) > 0) were analyzed for similarity based on their core enriched genes. To this end, the Jaccard index was calculated for all pairs of gene sets. The resulting similarity matrix was visualized as an undirected weighted network using the igraph package (v2.0.3). Nodes in the network represented gene sets, with edge weights corresponding to their Jaccard index values. Nodes were arranged using the force-directed layout algorithm by Fruchterman and Reingold, and colors were assigned based on the NES values. Proteins were further annotated as surface proteins if they were contained in the cardiac surfaceome database^23^.

To assess the contribution of different factors (individual, workflow, fixation) to the variation in the dataset, variance partitioning was performed with the variancePartition package^45^ (v1.34.0). Briefly, a mixed-effects model was constructed, and the ‘fitExtractVarPartModel’ function was used to estimate the proportion of variance explained. Partial least squares discriminant analysis was performed using the ‘plsda’ function from the mixOmics package^46^ (v6.28.0), with individual as the grouping variable.

### dda-PASEF measurements for library generation

Peptides were separated on a 25 cm Aurora column (IonOpticks) using an EASY-nLC (Thermo Fisher Scientific) UHPLC. Peptides were loaded in buffer A (0.1% formic acid) and separated using a 90 min stepped gradient of 2–21% solvent B (0.1% formic acid in 80% ACN) for 56 min, 21–31% solvent B for 21 min and 31–44% solvent B for 13 min, using a constant flow rate of 400 nl min^−1^. Column temperature was controlled at 50 °C. Upon elution, peptides were injected via a CaptiveSpray source and 20 μm emitter into a timsTOF pro mass spectrometer (Bruker) operated in PASEF mode. MS data were collected over a 100–1,700 *m*/*z* range with trapped ion mobility spectrometry (TIMS) mobility range of 0.6–1.6 1/K_0_. Ion mobility was calibrated using three Agilent ESI-L Tuning Mix ions 622.0289, 922.0097 and 1221.9906. Per cycle, 10 PASEF ramps (100 ms TIMS ramp, 100 ms accumulation time) were recorded for a total cycle time of 1.17 s. MS/MS target intensity and intensity threshold were set to 20,000 and 2,500, respectively. An exclusion list of 0.4 min for precursors within 0.015 *m*/*z* and 0.015 V cm^−2^ width was activated.

### Library generation from DDA data

Spectral libraries were generated using the FragPipe computation platform (v22) with MSFragger (v4.1), Philosopher (v5.1.1) and EasyPQP (v0.1.49). Raw dda-PASEF files were processed for peptide identification from MS/MS spectra by searching against a reviewed human proteome database (UP000005640, downloaded on 26 June 2024), which included common contaminants and decoy sequences. The MSFragger search engine was used with default search parameters. Deep learning-based scoring was applied to the output through MSBooster (v1.2.31), followed by rescoring with Percolator and protein inference using ProteinProphet. Philosopher then performed FDR filtering, and spectral libraries were constructed with EasyPQP. To this end, retention time alignment to a common indexed retention time (iRT) scale was performed using common internal retention time (ciRT) peptides, and ion mobility alignment automatically selected one run as the reference.

### dia-PASEF measurements

Peptides were separated on an Aurora (Gen3) 25 cm, 75 μM ID column packed with C18 beads (1.6 μm) (IonOpticks) using a Vanquish Neo (Thermo Fisher Scientific) UHPLC. Peptide separation was performed using a 90 min stepped gradient of 2–17% solvent B (0.1% formic acid in ACN) for 56 min, 17–25% solvent B for 21 min and 25–35% solvent B for 13 min, or a 45 or 101 min gradient, using a constant flow rate of 400 nl min^−1^. Column temperature was controlled at 50 °C. Upon elution, peptides were injected via a CaptiveSpray source and 20 μm emitter into a timsTOF HT mass spectrometer (Bruker) operated in dia-PASEF mode. MS data were collected over a 100–1,700 *m*/*z* range. The standard’long-gradient’ dia-PASEF method was used, which included 16 dia-PASEF scans with two 25 Da windows per ramp, a total mass range of 400–1,201 Da and a mobility range of 1.43–0.6 1/K_0_. The collision energy was decreased linearly from 59 eV at 1/K_0_ = 1.6 to 20 eV at 1/K_0_ = 0.6 V cm^−2^. Both accumulation time and PASEF ramp time were set to 100 ms. The total cycle time was 1.8 s.

### Library generation for SAN and ACM datasets

Pseudo-MS/MS spectra were generated from dia-PASEF files for DDA-like database searching using diaTracer (v1.1.5) integrated into FragPipe (v22). Peptide-spectrum matching was then performed together with dda-PASEF files using MSFragger with default settings, followed by rescoring with MSBooster and Percolator, protein inference with ProteinProphet and FDR filtering by Philosopher. The spectral library was generated using EasyPQP. The final libraries contained the following: 4,546 proteins and 90,631 precursors (SAN versus RA); 6,222 proteins and 101,143 precursors (ACM versus Donor).

### Raw data processing (dia-PASEF)

Single-shot dia-PASEF data were analyzed using DIA-NN v1.9, either against project-specific libraries generated with FragPipe or against an in silico library with deep learning-based predictions of retention time, ion mobility and spectra (library-free). The mass accuracy was fixed to an average value based on DIA-NN’s recommended settings for each experiment. In addition, the ‘Match between Run’ feature was enabled. For library-free searches, proteotypicity was inferred at the gene level. The DIA-NN output tables were filtered at 1% precursor and protein *q* values.

### Bioinformatics of label-free DIA data

#### Pre-processing

Quantitative analyses were performed using protein abundances inferred from unique peptides. Intensities were log_2_ transformed, and contaminants were removed. To reduce sparsity, features were retained only if they had at least 10 valid values (ACM versus donor dataset) or a minimum of three valid values (SAN versus RA dataset) in at least one condition. Imputation was applied exclusively to the SAN versus RA dataset after filtering for proteins with sufficient valid observations in one group, selectively replacing missing values in the other group with values drawn from the lower tail of the intensity distribution (downshift of 1.8, width of 0.3).

#### Statistical testing of differential protein abundance

Proteins were tested for differential abundance by fitting linear models to the log-transformed intensities using the ‘lmFit’ function from the limma package^47^ (v3.60.4). For SAN samples, where paired SAN and RA tissue was collected from each individual, the correlation structure was accounted for by specifying the block argument and estimating the intra-block correlation with the ‘duplicateCorrelation’ function. Moderated *t*-statistics were computed with the ‘eBayes’ and ‘topTable’ functions, applying empirical Bayes moderation of residual variances towards a global trend (limma-trend method^48^). *P* values were adjusted for multiple comparisons using the Benjamini–Hochberg method. For the ACM comparisons, differential expression analysis was performed both with and without including covariates (age, sex) in the linear model, to evaluate their influence on the disease-associated proteomic signature. In addition, when comparing the ACM cases to the larger control cohort, potential batch effects were accounted for by including experimental batch as a covariate in the linear model.

#### Cell-type inference

To predict the likely cell-type origin of proteins, a single-nucleus RNA sequencing dataset was used^24^. Log-normalized .h5ad files were read into Python (v3.9.19) using scanpy (v1.10.3), and the dataset was subset to include only single nuclei isolated from either the SAN or the ventricle. For each cell-type annotation provided, the average gene expression was calculated using numpy (v2.0.2). For the SAN subset, cell-type annotations were refined by assigning SAN_P cells as a distinct cell type (previously labelled under atrial cardiomyocytes). In addition, the fraction of cells expressing each gene (expression > 0) was calculated for all cell types. The resulting tables were imported into R for marker detection, following our previously established method^49^. First, genes from the single-nucleus RNA sequencing dataset were matched to their corresponding proteins in the proteomics dataset and filtered out if they were not quantified in both data sets. *Z*-scores were calculated for the remaining transcripts across cell types to determine cell-type specificity. Genes were considered markers if their *z*-score exceeded 2 (97.72th percentile) for nuclei from ventricular tissue or 2.5 (99.38th percentile) for SAN nuclei. Furthermore, a gene was required to be expressed in at least 25% of cells within a given cell type to qualify as a marker. Proteins in the proteomics dataset were ranked by *t*-statistic (condition A versus condition B). Each gene set, representing a cell type and its associated markers, was then tested for significant overrepresentation at the top or bottom of the ranked list of proteins using GSEA, as implemented in the ‘GSEA’ function from the clusterProfiler package. The analysis was performed with 10,000 permutations, and *P* values were adjusted for multiple comparisons (reporting both *q* values and Benjamini–Hochberg-adjusted *P* values).

#### Functional enrichment analysis

GSEA of gene ontology cellular components was performed as described above, with the exception that the minimum gene set size was set to 20 and that significant results were filtered using the ‘simplify’ function from the clusterProfiler package, applying a similarity cut-off of 0.7 to remove redundancy of the enriched terms. For finding community structure (clusters or modules of terms), the ‘cluster_louvain’ function from the igraph package was used.

Overrepresentation analysis was performed using the ‘compareCluster’ function from the clusterProfiler package, utilizing universal enrichment analyzer and Reactome pathway collections obtained through the msigdbr package^50^ (v7.5.1) with the background set to all proteins measured in the respective dataset.

### Immunohistochemsitry

Immunohistochemistry was performed using an anti-Collagen VI antibody (EPR17072) (ab182744, Abcam), raised in rabbit. Sections of FFPE human heart tissue (4 µm thick) were deparaffinized in Tissue Clear, rehydrated through graded alcohols and rinsed in tap water. Antigen retrieval was performed by boiling the sections in tris–EDTA–glycine (TEG) buffer (pH 9) in a microwave oven for 15 min. Slides were then blocked in 2% bovine serum albumin for 10 min, followed by overnight incubation at 4 °C with the primary antibody (1:500 in 2% bovine serum albumin). The following day, slides were incubated with a biotinylated goat anti-rabbit secondary antibody (BA-1000, Vector Laboratories) at a dilution of 1:200 for 40 min. Endogenous peroxidase activity was blocked using 3% hydrogen peroxide. Signal was amplified using Elite ABC Kit (PK-6100, Vector Laboratories) for 30 min and visualized with 3,3′-diaminobenzidine (DAB+) (SK-4105, Vector Laboratories) for 15 min. Counterstaining was performed with Mayer’s hematoxylin. Slides were dehydrated and mounted using Pertex mounting medium, then scanned using an Axioscan 7 slide scanner (Zeiss). Digital whole-slide images were analyzed with QuPath v0.5.1 to quantify DAB signal intensity within defined regions of interest corresponding to the SAN and adjacent atrial myocardium. For each region of interest, the mean DAB optical density was quantified as a proxy for protein expression. Data were exported for statistical analysis in R.

### Quantification of fibro-fatty myocardial replacement

Histological evaluation of myocardial tissue was performed on H&E-stained sections to quantify fibro-fatty replacement morphometrically as recommended in clinical setting^51^. Digital images of the stained sections were analyzed using ImageJ (v1.54p) software, with a systematic square grid superimposed over each image. Each grid intersection corresponded to an area of 4,000 μm^2^ giving a spacing of 62.25 μm in line with methodological studies on cardiac histology^52,53^. At every intersection point, the underlying tissue was classified as myocardium, adipose tissue or fibrosis. The relative proportion of each component was determined by dividing the number of classified points by the total number of intersections assessed. Data were imported into R and visualized using the ggtern package (v3.5.0).

### Statistics and reproducibility

Sample size was determined by the availability of human heart specimens, together with estimates from proteome data acquired from freshly collected and snap-frozen human heart samples; no formal sample size calculations were performed. For each experimental condition, a minimum of three replicates was included to allow statistical evaluation. One raw file from the diaPASEF analysis of FFPE tissue was excluded based on quality control criteria. Samples were randomized during preparation, and investigators were blinded to group labels at this stage. Blinding was not possible during macrodissection of anatomical subregions due to the inherent visibility of tissue morphology.

### Reporting summary

Further information on research design is available in the Nature Portfolio Reporting Summary linked to this article.

## Supplementary information

Reporting Summary

Supplementary Table

## Source data

Source Data Fig. 2 Statistical source data.

Source Data Fig. 3 Statistical source data.

Source Data Fig. 4 Statistical source data.

Source Data Fig. 5 Statistical source data.

Source Data Fig. 6 Statistical source data.

Source Data Extended Data Fig. 1 Statistical source data.

Source Data Extended Data Fig. 2 Statistical source data.

Source Data Extended Data Fig. 3 Statistical source data.

Source Data Extended Data Fig. 4 Statistical source data.

Source Data Extended Data Fig. 5 Statistical source data.

Source Data Extended Data Fig. 6 Statistical source data.

Source Data Extended Data Fig. 7 Statistical source data.

Source Data Extended Data Fig. 8 Statistical source data.

## Data Availability

Source data are provided with this paper. The mass spectrometry proteomics data have been deposited to the ProteomeXchange Consortium via the Proteomics Identifications (PRIDE)^54^ partner repository with the dataset identifier PXD060216. Pre-processed spatial/snRNA-sequencing data were downloaded from the heart cell atlas v2 (heartcellatlas.org).

## Code availability

Data analysis was performed using available packages, which are detailed in the Methods.
