# Supplementary material for: Quantitative proteomics of formalin-fixed, paraffin-embedded cardiac specimens uncovers protein signatures of specialized regions and patient groups
Source: Nat Cardiovasc Res. 2025 Sep 26;4(10):1409–23. doi: 10.1038/s44161-025-00721-2 (PMC12520988; doi:10.1038/s44161-025-00721-2)
Supplement: Supplementary file 1 — Reporting Summary [file 44161_2025_721_MOESM1_ESM.pdf]

nature portfolio

Corresponding author(s): Alicia Lundby

Last updated by author(s): Aug 14, 2025

Reporting Summary

Nature Portfolio wishes to improve the reproducibility of the work that we publish. This form provides structure for consistency and transparency in reporting. For further information on Nature Portfolio policies, see our Editorial Policies and the Editorial Policy Checklist.

Statistics

For all statistical analyses, confirm that the following items are present in the figure legend, table legend, main text, or Methods section.

n/a	Confirmed	
☐	☒	The exact sample size (*n*) for each experimental group/condition, given as a discrete number and unit of measurement
☐	☒	A statement on whether measurements were taken from distinct samples or whether the same sample was measured repeatedly
☐	☒	The statistical test(s) used AND whether they are one- or two-sided<br>*Only common tests should be described solely by name; describe more complex techniques in the Methods section.*
☐	☒	A description of all covariates tested
☐	☒	A description of any assumptions or corrections, such as tests of normality and adjustment for multiple comparisons
☐	☒	A full description of the statistical parameters including central tendency (e.g. means) or other basic estimates (e.g. regression coefficient) AND variation (e.g. standard deviation) or associated estimates of uncertainty (e.g. confidence intervals)
☐	☒	For null hypothesis testing, the test statistic (e.g. *F*, *t*, *r*) with confidence intervals, effect sizes, degrees of freedom and *P* value noted<br>*Give P values as exact values whenever suitable.*
☒	☐	For Bayesian analysis, information on the choice of priors and Markov chain Monte Carlo settings
☐	☒	For hierarchical and complex designs, identification of the appropriate level for tests and full reporting of outcomes
☐	☒	Estimates of effect sizes (e.g. Cohen's *d*, Pearson's *r*), indicating how they were calculated

Our web collection on statistics for biologists contains articles on many of the points above.

Software and code

Policy information about availability of computer code

Data collection	FragPipe (v20 and v22)<br>DIANN (v1.9)<br>QuPath (v0.5.1)
Data analysis	RStudio (2024.04.2, R v4.4.1) - pheatmap (v1.0.12), clusterProfiler (v.4.12.1), igraph (v.2.0.3), varianceParition (v1.34.0), mixOmics (v6.28.0), ggplot2 (v3.5.1), limma (v3.60.4), msigdbr (v7.5.1), ggtern (v3.5.0)<br>Python (v3.9.19) - scanpy (v1.10.3), numpy (v2.0.2)

For manuscripts utilizing custom algorithms or software that are central to the research but not yet described in published literature, software must be made available to editors and reviewers. We strongly encourage code deposition in a community repository (e.g. GitHub). See the Nature Portfolio guidelines for submitting code & software for further information.

Data

Policy information about availability of data

All manuscripts must include a data availability statement. This statement should provide the following information, where applicable:

- Accession codes, unique identifiers, or web links for publicly available datasets
- A description of any restrictions on data availability
- For clinical datasets or third party data, please ensure that the statement adheres to our policy

Source data are provided with the manuscript. The mass spectrometry proteomics data have been deposited to the ProteomeXchange Consortium via the PRIDE[45] partner repository with the dataset identifier PXD060216. Processed sc/snRNA-sequencing data was downloaded from the heart cell atlas v2 (heartcellatlas.org).

Research involving human participants, their data, or biological material

Policy information about studies with human participants or human data. See also policy information about sex, gender (identity/presentation), and sexual orientation and race, ethnicity and racism.

Reporting on sex and gender	Information on sex has been collected for all human FFPE tissue samples and is provided.
Reporting on race, ethnicity, or other socially relevant groupings	None reported
Population characteristics	Postmortem left ventricular human heart samples were collected from five individuals with non-cardiac causes of death.<br>Samples from the conduction-system were obtained following international guidelines during forensic and clinical autopsies performed at the Department of Pathology, Rigshospitalet.<br>Archival FFPE endomyocardial biopsy samples were sourced from the Department of Pathology, Rigshospitalet.
Recruitment	Samples were collected retrospectively.
Ethics oversight	Proteomic studies of human heart samples were carried out in agreement with Danish Law (Medical Research Involving Human Subjects Act) and institutional guidelines with ethical approval from The Regional Committee on Research Ethics (H-22012592). The data presentation is in accordance with the EU GDPR act 6(1) regarding public interest and 9(2)(j) regarding processing of special categories of personal data.

Note that full information on the approval of the study protocol must also be provided in the manuscript.

Field-specific reporting

Please select the one below that is the best fit for your research. If you are not sure, read the appropriate sections before making your selection.

☒ Life sciences ☐ Behavioural & social sciences ☐ Ecological, evolutionary & environmental sciences

For a reference copy of the document with all sections, see nature.com/documents/nr-reporting-summary-flat.pdf

Life sciences study design

All studies must disclose on these points even when the disclosure is negative.

Sample size	No sample size calculation was performed. A minimum of three replicates were included for each experimental condition.
Data exclusions	One raw file was excluded from the diaPASEF analysis of FFPE tissue as it was flagged as an outlier during quality control checks.
Replication	The reproducibility of findings was confirmed through inclusion of biological replicates (minimum of three required) and technical replicates where feasible. All attempts at replication were successful.
Randomization	Samples were randomized during sample preparation.
Blinding	Investigators were blinded to the group labels during sample preparation.<br>Blinding was not possible during macrodissection of anatomical subregions due to the nature of the tissue preparation.

Reporting for specific materials, systems and methods

We require information from authors about some types of materials, experimental systems and methods used in many studies. Here, indicate whether each material, system or method listed is relevant to your study. If you are not sure if a list item applies to your research, read the appropriate section before selecting a response.

Materials & experimental systems

n/a	Involved in the study
☐	☒ Antibodies
☒	☐ Eukaryotic cell lines
☒	☐ Palaeontology and archaeology
☒	☐ Animals and other organisms
☒	☐ Clinical data
☒	☐ Dual use research of concern
☒	☐ Plants

Methods

n/a	Involved in the study
☒	☐ ChIP-seq
☒	☐ Flow cytometry
☒	☐ MRI-based neuroimaging

Antibodies

Antibodies used

Anti-Collagen VI antibody [EPR17072] (AB182744-1001, Abcam)
Lot: 1030735-27

Validation

WB: Human skeletal muscle, Human placenta, Human fetal brain, Human fetal heart, Human fetal kidney, Human fetal spleen, Mouse heart, Mouse kidney, Mouse spleen, Rat kidney and Rat spleen lysates; HEK293T, WI-38 and NIH/3T3 whole cell lysates. IHC-P: Human liver, Human cardiac muscle, Mouse kidney and Rat stomach tissues. mIHC: Human liver and Human prostate gland tissues, human breast. ICC/IF: HeLa cells.

Plants

Seed stocks

Report on the source of all seed stocks or other plant material used. If applicable, state the seed stock centre and catalogue number. If plant specimens were collected from the field, describe the collection location, date and sampling procedures.

Novel plant genotypes

Describe the methods by which all novel plant genotypes were produced. This includes those generated by transgenic approaches, gene editing, chemical/radiation-based mutagenesis and hybridization. For transgenic lines, describe the transformation method, the number of independent lines analyzed and the generation upon which experiments were performed. For gene-edited lines, describe the editor used, the endogenous sequence targeted for editing, the targeting guide RNA sequence (if applicable) and how the editor was applied.

Authentication

Describe any authentication procedures for each seed stock used or novel genotype generated. Describe any experiments used to assess the effect of a mutation and, where applicable, how potential secondary effects (e.g. second site T-DNA insertions, mosiacism, off-target gene editing) were examined.
